# The sting in the tail of severe falciparum malaria: Post-artesunate delayed haemolysis

**DOI:** 10.4102/sajid.v37i1.392

**Published:** 2022-05-25

**Authors:** Yael Benjamin, David Stead

**Affiliations:** 1 Department of Internal Medicine, Frere Hospital, East London, South Africa; 2 Department of Medicine, Faculty of Health Sciences, Walter Sisulu University, Mthatha, South Africa

**Keywords:** malaria, zoonoses, protozoan infections, falciparum malaria, tropical diseases, haemolytic anaemia, autoimmune haemolytic anaemia

## Abstract

Post-artesunate delayed haemolysis (PADH) is thought to occur because of delayed clearance of previously malarial infected erythrocytes spared by ‘pitting’ during treatment. We report a case of PADH following the treatment of *Plasmodium (P.) falciparum* malaria (32% parasitaemia), with a positive direct antiglobulin (DAT) test, suggesting an immune mechanism.

## Background

An initial fall in haemoglobin (Hb), after starting antimalarial therapy, is common; however, this usually resolves within a week.^1^ The two main causes for this falling Hb are lysis of parasitised red cells (with splenic clearance) and concurrent suppression of haemopoiesis through inflammatory cytokines such as interleukin 6 (IL-6).^2,3^ Delayed onset haemolytic anaemia after parasite clearance has been described as a rare complication of severe *P. falciparum* malaria prior to the introduction of artemisinin therapies.^4^ More recently, post-artesunate delayed haemolysis (PADH) has been described following treatment with intravenous (IV) artemisinin (artesunate), occurring typically 1–3 weeks after treatment initiation in non-immune travellers.^4^ Treatment with intravenous artesunate is the recommended first line therapy for severe *P. falciparum* malaria in adults and children, with significant reductions in mortality compared to quinine (risk ratios [RR]: 0.61, 95% confidence interval [CI]: 0.50–0.75 and RR: 0.76, 95% CI: 0.65–0.90, respectively).^5^ Intravenous artesunate should be given for at least 24 hours; thereafter, once orals are tolerated, a transition can be made to oral artemisinin.^6,7,8,9^

## Case report

A 43-year-old man with no significant previous medical history was admitted to our institution with severe *P. falciparum* malaria.^2^ He originated from the low malaria risk area of Lahore, North-Eastern Pakistan, flew to Malawi and then travelled by road through Mozambique into South Africa, arriving in East London, Eastern Cape, three weeks later. He had not taken any malaria chemoprophylaxis prior to or during his travels. After a week of progressive fever and headache, he was referred to the Frere hospital emergency unit. The patient was found to be acutely ill. He was confused, dehydrated, jaundiced, pyrexial (39 °C), tachypnoeic (34 breaths per min), tachycardic (115 beats per min), and his blood pressure was within normal ranges (135/80). He was generally weak, with no signs of meningism, and had moderate generalised abdominal tenderness but was not peritonitic. He had no palpable liver or spleen on examination and other systems were otherwise unremarkable.

He was resuscitated with 1 L of intravenous normal saline (1L IV N/S) during his first hour in the emergency unit and, thereafter, received 1L IV N/S eight hourly. Urgent blood tests were taken and sent to the lab. A full blood count (FBC) revealed an Hb of 11 g/dL (normal range: 13–17), a normal leucocyte count, a reduced platelet count of 35 × 10^9^/L (normal range: 186–454) and a reticulocyte production index (RPI) of 0.4 (normal range: 1–2). Serum chemistry revealed: creatinine – 185 mmol/L, sodium – 129 mmol/L, total bilirubin – 165 umol/L, conjugated bilirubin – 93 umol/L, haptoglobin – 0.03 g/L, lactate dehydrogenase (LDH) – > 2700 U/L and C-reactive protein (CRP) – 306 mg/L. The patient tested positive for *P. falciparum* malaria on a rapid antigen test (immunocapture [ICT] Malaria *P.f* Antigen rapid diagnostic test [RDT]) and IV artesunate was commenced. The patient weighed 72 kg and 175 mg of IV artesunate was administered at 0, 12, 24 and 48 h according to guidelines – (2.4 mg/kg per dose). The thin blood smear confirmed *P. falciparum* intra-erythrocytic parasites, with a parasite count of 32%. This, together with the clinical picture, classified the malaria as severe. The patient was admitted to a general ward and was managed primarily by a nephrologist, together with the input of an infectious diseases specialist.

**FIGURE 1:**
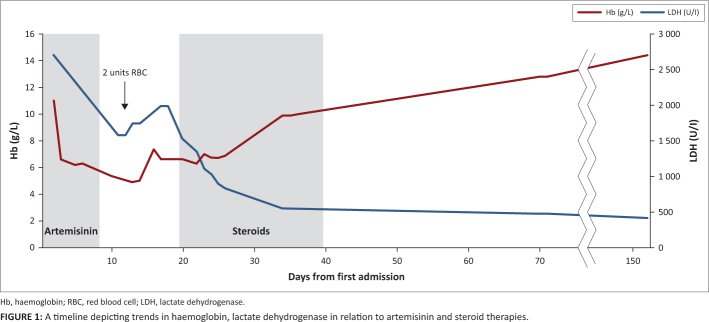
A timeline depicting trends in haemoglobin, lactate dehydrogenase in relation to artemisinin and steroid therapies.

During admission, the patient was closely monitored both clinically and by means of blood investigations (see Table 1), including the following: FBC, renal function, electrolytes, LDH, reticulocyte count, liver function tests (LFTs) and thin blood smears. In the first week of admission, renal function, LDH and Hb were monitored daily. By day 3 of admission, the patient’s fever defervesced, jaundice had improved, thin blood smear revealed that parasitaemia had decreased to 1% and platelets had improved to 60 × 10^9^/L; however, the Hb had decreased to 6.6 g/dL. A repeat smear on day 4 revealed no malaria parasites (confirmed by two haematology technologists). On day 5, IV artesunate was converted to three days of oral artemether/lumefantrine. A peak serum creatinine of 753 umol/L was recorded on day 7 of admission but steadily improved thereafter without requiring haemodialysis. By day 11, the Hb had continued to drop (5.1 g/dL) with no clear explanation. A repeat blood smear was ordered and confirmed the absence of malaria parasites. On day 13, the patient’s Hb was 4.9 g/dL and he had 2 units of packed red cells (PRC) transfused to a post transfusion Hb of 5.1 g/dL. In the week following the transfusion, the Hb increased minimally to 6.3 g/dL but did not increase further. There was evidence of ongoing haemolysis with LDH remaining at 1990 U/L on day 18 and a positive direct antiglobulin (DAT) with predominantly immunoglobulin G (IgG) activation indicating warm antibody involvement. Other secondary causes of autoimmune haemolytic anaemia (AIHA) were negative on testing (anti-nuclear antibody, HIV serology, Treponema pallidum haemagglutination assay and viral hepatitis screen). Non-immune haemolysis because of glucose-6-phosphate dehydrogenase (G6PD) deficiency was also excluded. Vitamin B12 and serum folate levels were within normal limits. The reticulocyte count was initially 0.93% with an RPI of 0.4; the count later increased to 8.4%, with an RPI of 0.9 on day 14. No bone marrow biopsy was performed by the attending team as it was not deemed essential for the patient’s work-up. The working diagnosis was a drug induced AIHA secondary to artesunate and, despite only anecdotal evidence of benefit, oral prednisone at 1 mg/kg daily was initiated on day 21. By day 22, haemoglobin had improved slightly to 6.6 mmol/dL, renal and LFT were normal and the patient was discharged on day 25. He was seen as an outpatient after 2 weeks of corticosteroids (CS) with an Hb of 9.9 g/d and an LDH of 550 U/L. Prednisone was then rapidly weaned over the next 2 weeks and his Hb continued to normalise to the point of discharge on day 152. At this time, the patient had an Hb of 14.4 mmol/dL, LDH of 418 U/L and a negative DAT. He was clinically well (see Figure 1). There were no steroid complications evident.

**TABLE 1:** Trends in key haematology and biochemistry blood results.

Days from first admission	0	2	4	6	7	10	11	13	14	15	18	20	21	22	23	24	34	39	152
Parasitaemia %	32%	1%	Nil	-	-	-	Nil	Nil	-	Nil	Nil	-	-	-	-	-	-	-	-
Hb (g/dL)	11%	7%	6.2	6.3	5.9	5.4	5.1	4.9*	5.1	7.4	6.6	6.3	6.3**	6.6	7	6.4	9.9	12.8	14.4
LDH (U/L)	> 2700	-	-	-	-	-	1577	1741	-	-	1990	1531	1348		1118	960	550	478	418
RPI	0.4	-	-	-	-	-	-	-	0.9	-	-	-	-	0.5	0.6	0.6	1.5	13	1.6
Na (mmol/L)	129	136	140	133	138	138	140	-	-	-	-	138	-	-	-	139	136	141	136
K (mmol/L)	4.4	4.2	4.1	4.2	4.5	4.4	5	-	-	-	-	5.1	-	-	-	3.5	3.7	4.4	4
Ur (mmol/L)	22.1	52.1	49.4	44.6	41	28.6	24.1	-	-	-	-	6	-	-	-	4.4	-	5.1	3.2
Cr (umol/L)	185	576	672	740	753	629	554	-	-	-	-	186	-	-	-	91	80	63	74
CRP (mg/L)	306	-	-	-	-	-	-	-	-	-	-	-	-	-	-	< 1	-	-	-
ALT (U/L)	28	-	-	-	-	-	-	-	-	-	14	-	-	-	-	23	-	-	16
	IV artemesinin	-	-	Coartem		-	-	*2 units RBC			-	-	**CS	-	-	-	-	-	

Hb, haemoglobin; LDH, lactate dehydrogenase; RPI, reticulocyte production index; Na, sodium; K, potassium; Ur, urea; Cr, creatinine: CRP, C reactive protein; ALT, alanine transaminase.

## Discussion

This is the first South African report of PADH, complicating severe *P. falciparum* infection with a high parasitaemia. There is no clear consensus definition for PADH, but most literature refer to a > 10% decrease in Hb or a > 10% increase in LDH occurring more than eight days after the initiation of treatment.^10^ As much as 22% of patients with severe malaria experience PADH and up to 50% of these require blood transfusions.^11,12^ Anaemia usually improves 4–8 weeks after the completion of artesunate therapy.

In this case, the patient presented in multi-organ failure, showing rapid clinical improvement following the initiation of IV artesunate. There was a rapid drop in parasitaemia from 32% to 1% after three days of IV artesunate and his clinical improvement aligned with this. Initial features of haemolysis with a rising LDH and falling haptoglobin were seen. There was also a poor reticulocyte response that later improved but remained below adequate response levels showing suppressed erythropoiesis as a contributing factor. Reduced erythropoiesis from malaria infection is well described as a contributor to malarial anaemia because of a number of mechanisms including cytokines, altered iron metabolism and haemozoin (malarial pigment by-product).^13^ There is also some evidence of artemisinin directly suppressing proerythroblast growth and differentiation.^1^

The mechanism of PADH is not fully understood but is thought to be because of delayed clearance of infected erythrocytes spared by ‘pitting’ during treatment with artesunate.^11^ ‘Pitting’ is a process that occurs in the spleen with artemisinin treatment, whereby dead ring-form parasites are expelled from their host erythrocytes without causing cell destruction. The erythrocytes then return into circulation with a reduced life span compared to non-parasitised erythrocytes. This is because of cellular damage and smaller surface area resulting from the pitting process.^11^ A high initial parasitaemia is associated with increased risk for severe PADH as more erythrocytes have undergone this ‘pitting’ process (as seen in this case with a parasitaemia of 32%). Quinine and other antimalarials do not share this pitting effect.^12^

There is some evidence for a drug-induced autoimmune component to PADH, in that at least 40% of 10 PADH cases from a single centre were DAT positive. Corticosteroids were given in three of the DAT positive patients leading to good outcomes.^10^ Le Brun et al.^14^ reported a single case of successful CS use in DAT positive PADH. Corticosteroids were started in the current case because of a dropping Hb, positive DAT and the suspicion of a warm antibody drug induced AIHA. Stabilisation and recovery of the haemoglobin did coincide with the initiation of CS, but this may have been a coincidence. There is a need for prospective randomised control trials to look at the potential transfusion sparing role of CS in such cases.

There are case reports of oral artemisinin therapy associated PADH; however, this is rarely of clinical significance as the drop in Hb is usually minimal. It is more commonly of clinical significance in patients treated with parenteral artemisinin therapy.^15^ With this risk of delayed onset anaemia, treatment with parenteral artesunate should be limited to the period for which it is required and a full oral course of an appropriate anti-malarial drug should be used thereafter.^6,11^

We believe that this case demonstrates PADH with an auto-immune component evidenced by the positive DAT. Oral CS were given and coincided with a steady recovery in the Hb. This is an important late complication of treating severe falciparum malaria with artesunate that clinicians should be aware of.

## Teaching points

- Severe *P. falciparum* malaria cases with high parasite counts are at risk of PADH and should be routinely monitored for this late complication.
- A dropping haemoglobin or rising LDH beyond 8 days from starting artesunate therapy may indicate PADH.
- The primary management of PADH is supportive. Corticosteroid use has been reported, but any randomised evidence of benefit is lacking.
